# PM014 attenuates radiation-induced pulmonary fibrosis via regulating NF-kB and TGF-b1/NOX4 pathways

**DOI:** 10.1038/s41598-020-72629-9

**Published:** 2020-09-30

**Authors:** Sung-Hyo Park, Jee-Youn Kim, Jin-Mo Kim, Byeong Rok Yoo, Song Yee Han, Yoo Jin Jung, Hyunsu Bae, Jaeho Cho

**Affiliations:** 1 Department of Radiation Oncology, Yonsei University College of Medicine, 50-1 Yonsei-ro, Seodaemun-gu, Seoul, 03722 Republic of Korea; 2 Department of Science in Korean Medicine, Graduate School, Kyung Hee University, Seoul, 02447 Republic of Korea; 3 Department of Physiology, College of Korean Medicine, Kyung Hee University, #1, Hoegi-dong, Dongdaemun-gu, Seoul, 130-701 Republic of Korea

**Keywords:** Molecular biology, Medical research, Molecular medicine

## Abstract

Radiation therapy is the mainstay in the treatment of lung cancer, and lung fibrosis is a radiotherapy-related major side effect that can seriously reduce patient’s quality of life. Nevertheless, effective strategies for protecting against radiation therapy-induced fibrosis have not been developed. Hence, we investigated the radioprotective effects and the underlying mechanism of the standardized herbal extract PM014 on radiation-induced lung fibrosis. Ablative radiation dose of 75 Gy was focally delivered to the left lung of mice. We evaluated the effects of PM014 on radiation-induced lung fibrosis in vivo and in an in vitro model. Lung volume and functional changes were evaluated using the micro-CT and flexiVent system. Fibrosis-related molecules were evaluated by immunohistochemistry, western blot, and real-time PCR. A orthotopic lung tumour mouse model was established using LLC1 cells. Irradiated mice treated with PM014 showed a significant improvement in collagen deposition, normal lung volume, and functional lung parameters, and these therapeutic effects were better than those of amifostine. PM104 attenuated radiation-induced increases in NF-κB activity and inhibited radiation-induced p65 translocation, ROS production, DNA damage, and epithelial-mesenchymal transition. PM104 effectively alleviated fibrosis in an irradiated orthotopic mouse lung tumour model while not attenuating the efficacy of the radiation therapy by reduction of the tumour. Standardized herbal extract PM014 may be a potential therapeutic agent that is able to increase the efficacy of radiotherapy by alleviating radiation-induced lung fibrosis.

## Introduction

Radiotherapy is one of the most important treatment strategies for thoracic tumours such as lung cancer, esophageal cancer, and breast cancer. However, thoracic radiation causes irradiation (IR)-induced pulmonary injury including pneumonitis and fibrosis, which affects the patients’ breathing and quality of life, and is the main cause of fatality in patients after lung cancer radiotherapy^1^. The consequence of radiation (IR)-induced pulmonary injury are typically divided into early radiation toxicity and late radiation toxicity. Thoracic radiation induced pneumonitis is early reversible toxicity and fibrosis is irreversible late toxicity. To date, the exact mechanisms involved in the development of fibrosis in response to irradiation therapy are not yet fully understood. At the cellular level, radiation injures epithelial and endothelial cells and induce inflammatory and profibrotic cytokines, including transforming growth factor‑β1 (TGF‑β1), tumour necrosis factor‑α (TNF‑α), interleukin‑1β (IL‑1β), and interleukin‑6 (IL‑6), which promotes the activation of fibroblasts. Activated fibroblasts and myofibroblast produce collagen and extracellular matrix proteins such as fibronectin which results in radiation-induced lung fibrosis. Through Epithelial-mesenchymal transition (EMT), alveolar epithelial cells (AECs) produce important source of myofibroblasts^2^ and may also undergo differentiation into myofibroblasts. EMT, which is characterized by acquisition and upregulation of mesenchymal biomarkers, including α-smooth muscle actin (α-SMA), vimentin and fibronectin, is reported to play an important role in fibrosis of various tissues including idiopathic lung fibrosis. Thus, it is important to understand the EMT process in development of radiation induced fibrosis, and the reverse mechanism of EMT is considered an effective strategy for prevention and treatment of radiation-induced pulmonary fibrosis.

DNA damage response (DDR) and apoptosis both play key roles in radiation-induced cell death regulation^3,4^, and the presence of apoptosis-resistant fibroblasts promotes progressive fibrosis. The NF-kB pathway is known to be activated following IR exposure^5^ and required for the induction and maintenance of EMT, and has been shown to affect cell survival and cause cells to become resistant to radiotherapy^6,7^. Therefore, suggesting agents that regulate the DNA damage response, apoptosis, and the activation of NF-κB may be effective therapeutic strategies for radiotherapy-induced pulmonary fibrosis.

Herbal medicines have been used for centuries in Asian countries to treat various diseases, and because of their ability to hit multiple targets, they could be a better choice as radioprotective agents. PM014 is a standardized herb extract derived from the herbal mixture Chung-Sang-Bo-Ha-Tang (CSBHT), which has been especially used to treat chronic lung diseases such as asthma and chronic obstructive pulmonary disease (COPD) in Traditional Korean Medicine. Recently, it is reported that anti inflammation and treatment effect of PM014 in lung diseases model including lipopolysaccharide (LPS)-induced acute lung inflammation, cigarette smoke-induced lung inflammation in chronic obstructive pulmonary disease (COPD), and cockroach allergen-induced asthma. Previously, we demonstrated the potent protective effect of PM014 in radiation-induced lung inflammation by regulating inflammasome^8^. Irradiation-induced lung damage is divided into early stage of latent phase, mid stage with acute pneumonia, and maturity with fibrosis according to clinical conditions, and there is a difference in pathologic changes^9,10^. As such, radiation pneumonia and pulmonary fibrosis were considered to be separate syndromes due to differences in the order of occurrence and target cells. However, this concept has been changed in recent studies. Each pulmonary reaction is in a continuous line without a clear division of temporal order, that is, due to the early activation of the inflammatory response, the cytokine cascade is expressed and sustained, resulting in late pulmonary fibrosis^11–13^. We developed a mouse model that simulates clinical stereotactic body radiotherapy (SBRT) using an image-guided animal irradiation system to deliver a single 75 Gy dose to the mouse lung^8^ and validated the induction of lung fibrosis following high-dose IR^14^. Using this model, in the present study, we focused on the role and mechanism of PM014 in radiation-induced lung fibrosis, and compared its effects to those of amifostine. We also investigated the effects of PM014 not only on preventing fibrosis in normal cells but also on inhibiting tumour cells including cancer stem-like cells.

## Results

### Effect of PM014 on gross anatomical and histopathological damage

**Figure 1:**
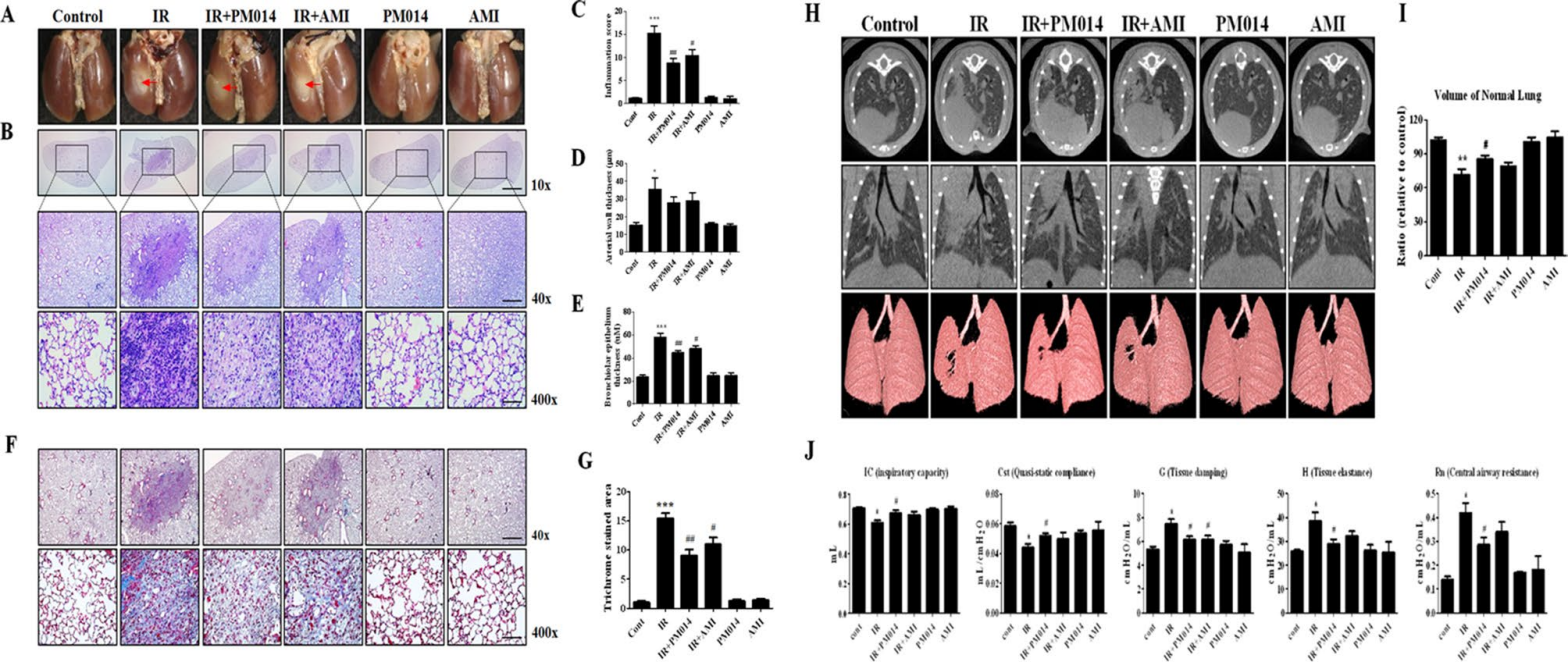
Effect of PM014 on lung fibrosis in irradiated mice. (**A**) Representative gross findings. (**B**) H&E-stained lung sections. Magnification, × 10 (Scale bar 4 mm), 40 × (Scale bar 1 mm), and 400 × (Scale bar 100 μm). Quantification of the inflammatory score (**C**), arterial wall thickness (**D**), and bronchiolar epithelium thickness (**E**). (**F**) Masson’s trichrome-stained lung sections. Magnification, × 40 (Scale bar 1 mm), and × 400 (Scale bar 100 μm). (**G**) Quantification of fibrotic area. Representative micro-CT images of mouse lungs. (**H**) Horizontal (top), trans-axial (middle), and 3D micro-CT (bottom). (**I**) Quantification of the volume of the normal left lung. (**J**) Functional measurements of mouse lungs. The treatment groups were Control (untreated); IR, (75 Gy irradiation); IR + PM014 (IR + 200 mg/kg PM014); IR + AMI (IR + 100 mg/kg amifostine); PM014 (200 mg/kg PM014 only); and AMI (100 mg/kg amifostine only).

We previously identified time dose response for fibrosis after high dose focal radiotherapy to the mouse lung and as a result, considered a 6th week model suitable for studying IR-induced lung fibrosis (Supplementary Fig. S1). To elucidate whether PM014 inhibited IR-mediated lung fibrosis, we compared changes in the surface morphology of the left lung in the control and IR groups at 6 weeks after irradiation with 75 Gy. In contrast to the control mice, the irradiated areas of the left lung clearly exhibited a local, white, ring-like injury (Fig. 1A), infiltration of inflammatory cells, and alveolar wall and bronchiolar epithelium thickening (Fig. 1B–E). To confirm fibrosis, Masson’s Trichrome staining was performed. At 6 weeks, intense blue stained collagen fibers were observed in the IR group (Fig. 1F,G). Oral administration of PM014 (200 mg/kg) on alternate days for 6 weeks after irradiation resulted in significantly less gross anatomical changes, histological damage, and fibrosis compared to the IR group. These protective effects of PM014 were better than those observed for amifostine (AMI, 100 mg/kg, i.p.).

### Micro-computed tomography and lung functional analysis

For image evaluation of IR-induced lung fibrosis, we used a non-invasive micro-computed tomography (CT), equivalent to clinical CT in humans^15^. Six weeks after irradiation, typical micro-CT manifestations of IR-induced lung injury^16^ were observed in the irradiated left lung (lighter grey). However, these effects were decreased in PM014-treated mice (Fig. 1H,I). The normal lung volume in the IR group was lower than that in control mice. However, in the IR + PM014 group, the volume was significantly recovered compared to that in the IR group (P < 0.05). PM014 was more effective than AMI (100 mg/kg) in maintaining normal lung volume. Radiation-induced changes in lung function were evaluated using the flexiVent system^17^. Compared to those in the control group, both inspiratory capacity (IC) and quasistatic compliance (Cst) in the IR group were significantly decreased, reflecting a reduced total capacity and stiffness of the lung. Both tissue damping (G) and tissue elastaince (H), parameters assessing lung tissue rigidity, were significantly increased in the IR group, suggesting lung parenchymal injury, and there was an increase in Newton resistance (Rn) in the IR group, indicating airway hyper-responsiveness (Fig. 1J). However, this IR-induced respiratory distress was significantly reduced in PM014-treated mice, suggesting that PM014 has a protective effect on IR-induced deterioration in lung function. These effects of PM014 were also more prominent than those observed for AMI (100 mg/kg).

### Inhibition of radiation-induced fibrosis-related molecules by PM014

**Figure 2:**
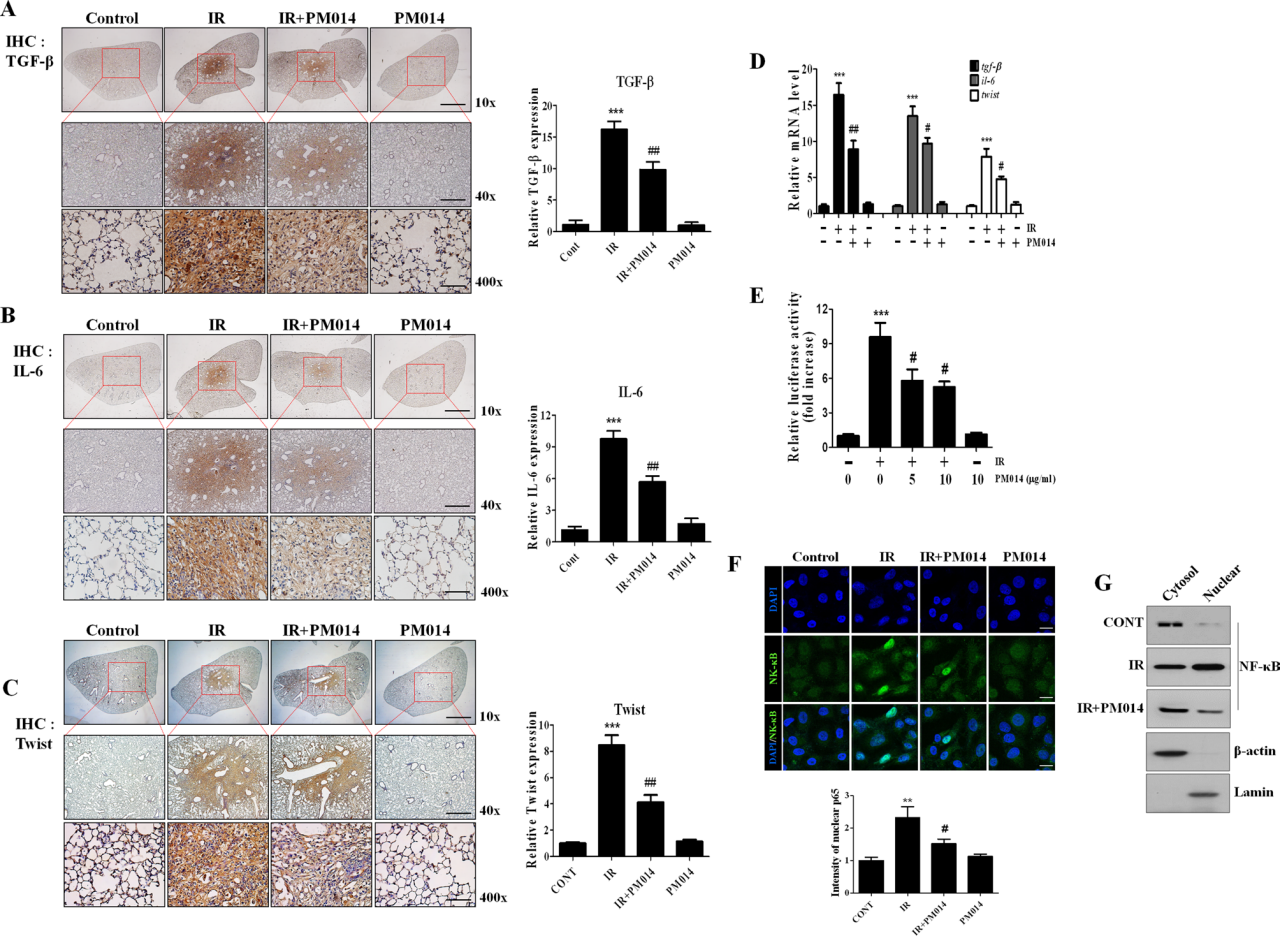
Effect of PM014 on the expression of fibrosis-related molecules and NF-κB activation. Immunohistostaining of TGF-β (**A**), IL-6 (**B**), and Twist (**C**). Quantification of the stained tissue is shown on the right. Magnification, × 10 (Scale bar 4 mm), × 40 (Scale bar 1 mm), and × 400 (Scale bar 100 μm). (**D**) Effect of PM014 on the mRNA levels from mouse lung tissue of *TGF-β*, *IL-6*, and *Twist*. (**E**) Activation of the NF-κB promoter by irradiation. After 24 h of transfection, the cells were subjected to 10 Gy irradiation for 6 h with or without the indicated dose of PM014 and luciferase activity was determined. (**F**) After 12 h of irradiation with 10 Gy, L132 cells were stained with an anti-p65 antibody to examine p65 nuclear translocation. Magnification, × 400 (Scale bar 20 μm). (**G**) Immunoblotting studies of NF-kB subcellular localization in irradiated L132 cells. Full length blots are presented in Supplementary Fig. S9.

To investigate the molecules responsible for radiation-induced lung fibrosis, a cDNA microarray analysis of lung tissues after focal exposure to 75 Gy was performed^18^. Of these genes, we focused on transforming growth factor-β (*TGF-β*), *IL-6*, and Twist (Supplementary Table S1), which are key molecules involved in fibrosis progression and stimulate collagen synthesis in fibroblasts and myofibroblasts^19,20^. Immunohistochemistry and RT-PCR revealed higher TGF-β1, IL-6, and Twist expression levels in irradiated lungs than in controls, which were attenuated by PM014 (Fig. 2A–D). We also investigated whether NF-κB is also regulated by IR and PM014 using an NF-κB promoter driving luciferase expression. Luciferase activity was increased 9.57-fold in irradiated cells compared to that in control cells (Fig. 2E); however, it was suppressed by 33.1% and 41% in PM014-treated cells (5 μg/mL and 10 μg/mL PM014, respectively). Moreover, nuclear translocation of p65 was increased by IR, and this was inhibited by PM014 treatment (Fig. 2F,G). These results suggest that NF-κB is activated by IR to increase the expression of downstream pro-fibrotic effectors, resulting in fibrosis.

### Inhibition of radiation-mediated epithelial-mesenchymal transition (EMT) features by PM014

**Figure 3:**
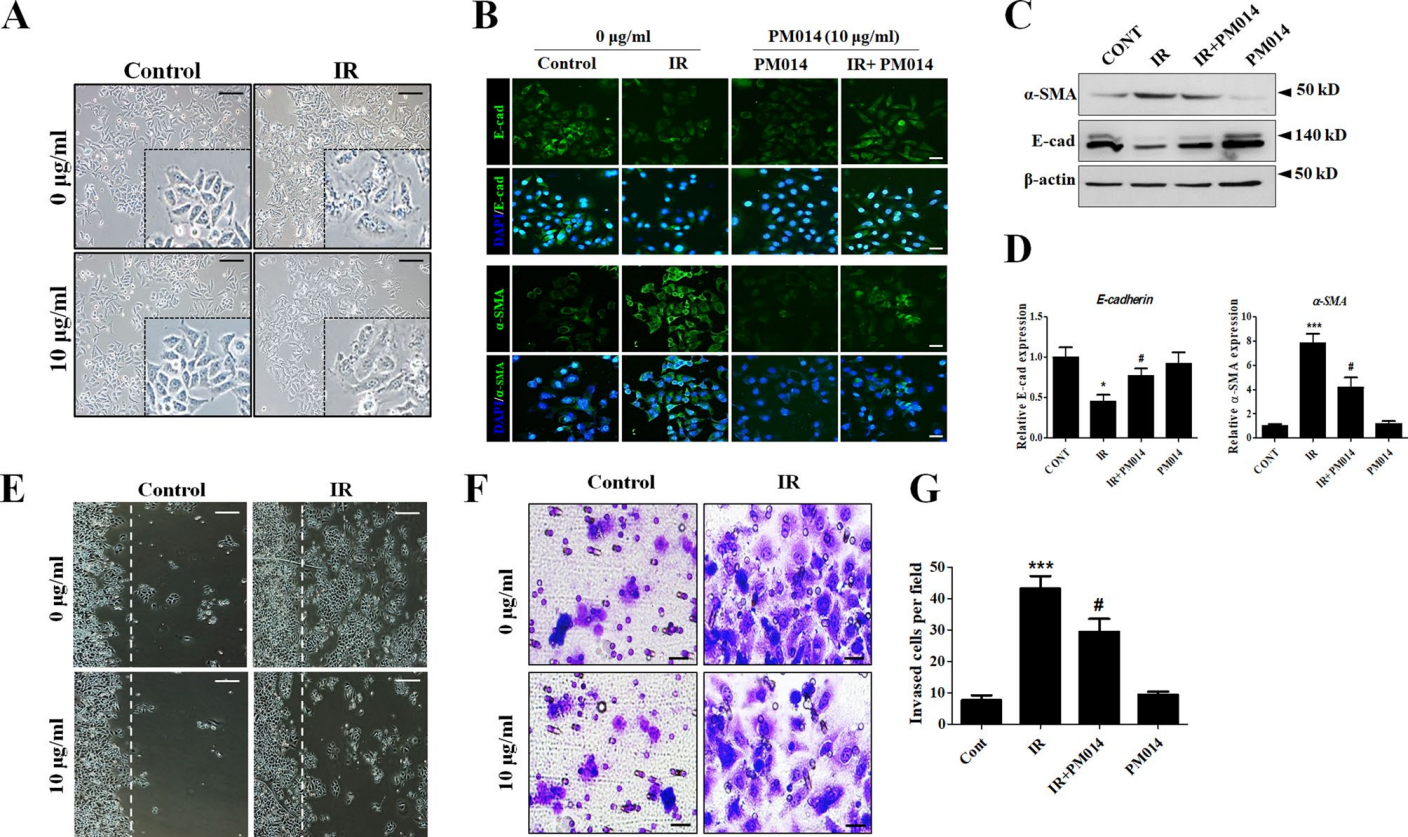
Effect of PM014 on IR-mediated EMT features. To analyze the induction of EMT by IR, L132 cells were irradiated with 10 Gy for 72 h with or without PM014 (10 μg/mL). (**A**) IR caused the L132 cells to adopt a spindle shape, which was attenuated by PM014 treatment. Magnification, × 100 (Scale bar 100 μm). (**B**) Irradiated L132 cells showed decreased levels of the epithelial cell marker (E-cad) and increased levels of the mesenchymal cell marker (α-SMA); however, PM014 reversed these phenomena. Magnification, × 200 (Scale bar 50 μm). Cell lysates were analyzed by western blotting. Full length blots are presented in Supplementary Fig. S9 (**C**) and RT-PCR (**D**). (**E**) Wound closure assay to assess the migratory capacity of L132 cells. Magnification, × 100 (Scale bar 100 μm). (**F**) L132 cells were plated into a Matrigel-coated trans-well invasion chamber and incubated for 48 h. Magnification, × 200 (Scale bar 50 μm). (**G**) Invading cells per field were counted.

During fibrosis, EMT allows epithelial cells to undergo morphological changes^21^. We hypothesized that PM014 affects the EMT-like process in epithelial cells caused by radiation-induced fibrosis. To explore this hypothesis, we first examined morphological changes in L132 human lung epithelial cells after IR exposure. Following exposure to IR, the cells underwent a morphological transformation adopting a spindle-like shape. However, PM014 treatment attenuated these IR-mediated morphological changes (Fig. 3A). Immunocytochemical analysis confirmed that IR decreased the expression of the epithelial marker E-cadherin, and increased that of the mesenchymal marker α-SMA. However, PM014 restored the altered expression of these EMT-related molecules induced by IR (Fig. 3B). Western blot and RT-PCR analysis of E-cadherin and α-SMA expression also showed a similar pattern to that observed in the immunocytochemical analysis (Fig. 3C,D). Cell migration was significantly increased by IR, whereas in PM014-treated cells, IR-induced cell migration was greatly reduced (Fig. 3E). We also measured the invasiveness of irradiated L132 cells, which was increased 5.7-fold compared to that of control cells. However, this increase in invasiveness was attenuated by PM014 treatment (3.9-fold compared to control).

These results suggest that irradiated cells undergo EMT and that PM014 attenuates the acquisition of these IR-induced EMT features.

### PM014 reduces radiation-induced oxidative stress and DNA damage

**Figure 4:**
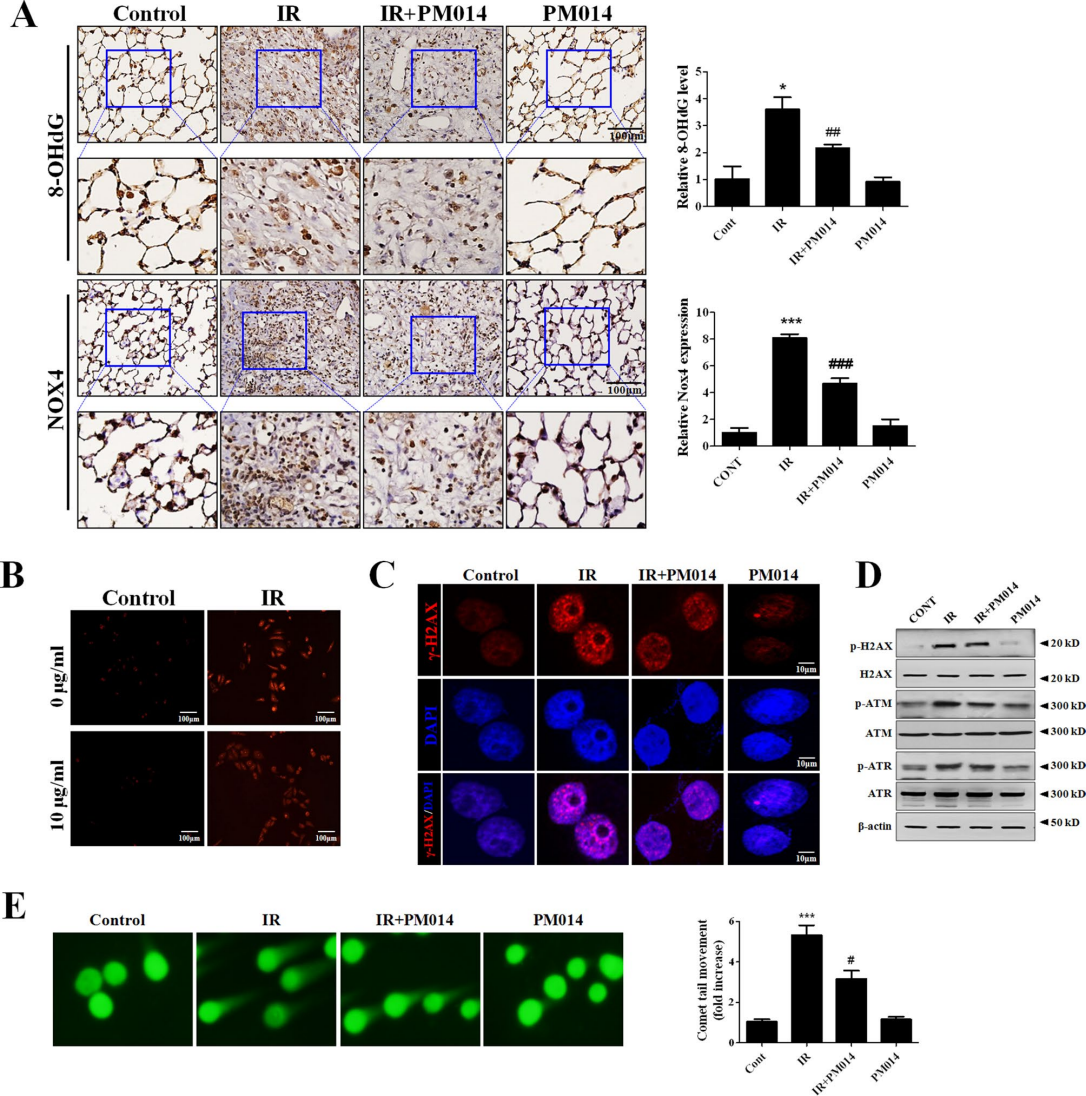
Effect of PM014 on IR-induced oxidative stress and DNA damage. (**A**) Immunohistochemistry to assess 8-OHdG and NOX4 levels was performed in mouse lung tissues. Quantification of stained tissue is shown on the right. Magnification × 400 (Scale bar 100 μm). L-132 cells were treated with 10 Gy for 6 h (b–e). (**B**) Fluorescence microscopic images of IR-induced ROS formation in L132 cells using MitoSOX red. Magnification × 100, Scale bar 100 μm. (**C**) Immunofluorescence staining with anti-phospho-H2AX (S139) antibody to detect radiation-induced DNA damage (Scale bar 10 μm). (**D**) Western blotting analysis of γ-H2AX, p-ATM, and p-ATR expression. Full length blots are presented in Supplementary Fig. S9. (**E**) Representative images of comet tail movement (left). The histograms display a quantification of the relative comet tail length, as measured by comet tail movement and normalized to control cells (right).

We assessed oxidative stress using immunohistochemistry for 8-OHdG and NOX4 in irradiated lung tissue. 8-OHdG and NOX4 levels were increased in the IR group, and these levels were significantly decreased by PM014 treatment (Fig. 4A), suggesting that PM014 has an antioxidant capacity. Due to the potential antioxidant capacity of PM014, we hypothesized that PM014 may have ROS scavenging activity as part of its radioprotective function. To verify this, we performed a DPPH radical scavenging assay. PM014 exhibited radical scavenging activity in a relatively dose-dependent manner (Supplementary Fig. S2). ROS formation was found to be markedly elevated in irradiated L-132 cells, whereas, in PM014-treated cells, there was a noticeable decrease in ROS formation (Fig. 4B). To investigate the effect of PM014 on IR-induced DNA damage, we measured H2AX phosphorylation (γH2AX) status by western blot and immunocytochemistry. This IR-induced stimulation of H2AX phosphorylation was reduced by PM014 (Fig. 4C). IR treatment resulted in a significant increase in γH2AX positive nuclei (Supplementary Fig. S3). The IR-induced phosphorylation of ATM and ATR was also decreased by PM014 (Fig. 4D, Supplementary Fig. S4). Irradiated cells had a significantly longer comet tail than PM014-treated cells (Fig. 4E). These findings suggest that radiation-induced DNA damage is prevented by PM014 treatment.

### PM014 attenuates IR-induced apoptosis in alveolar epithelial cells

**Figure 5:**
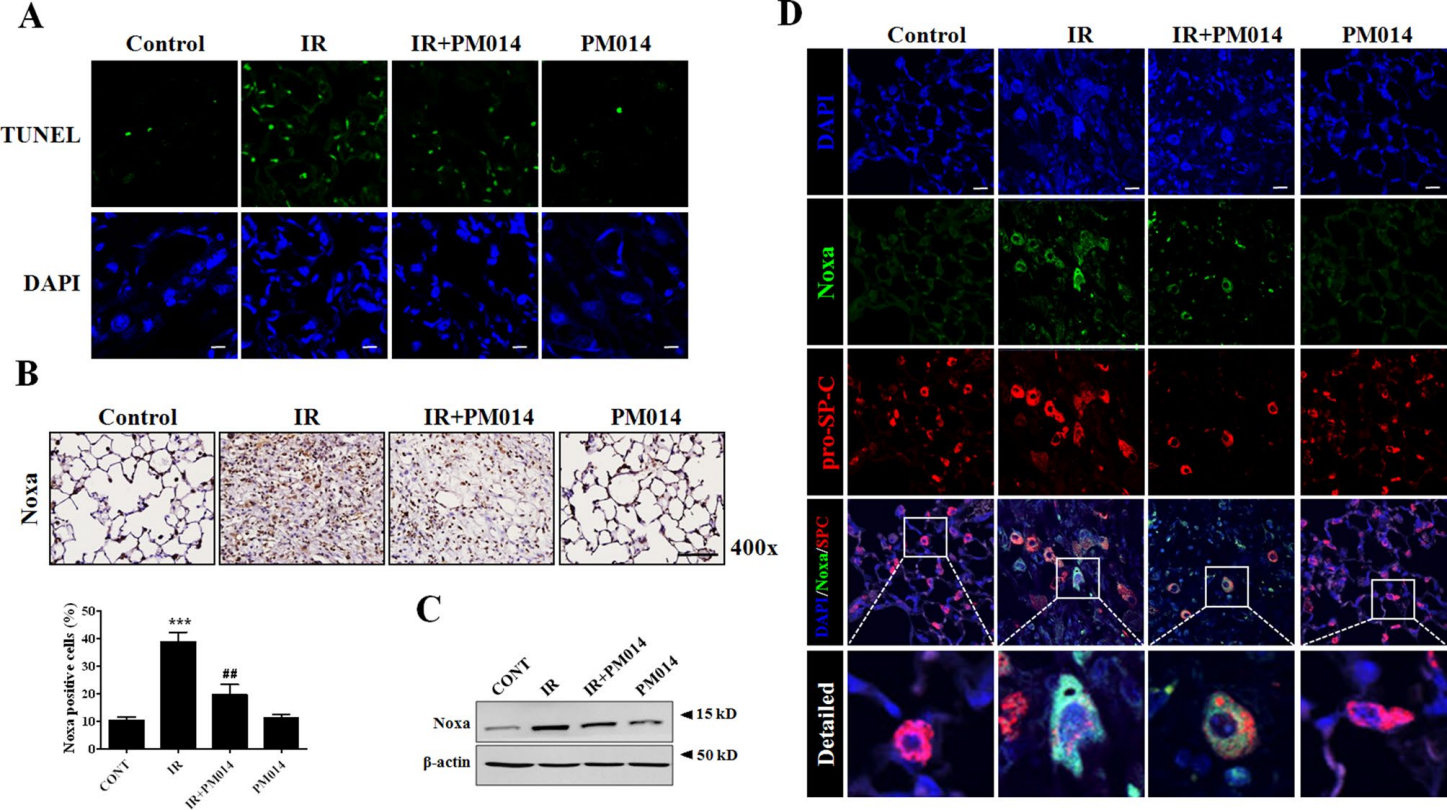
Effect of PM014 on IR-induced apoptosis. (**A**) Representative TUNEL staining (green) of irradiated mouse lung tissues. Magnification, × 400 (Scale bar 50 μm). The treatment groups were Control (untreated); IR, (75 Gy irradiation); IR + PM014 (IR + 200 mg/kg PM014); PM014 (200 mg/kg PM014 only). Immunohistochemistry (**B**) and western blotting (**C**) using irradiated mouse lung tissues. Magnification, × 400 (Scale bar 100 μm). Full length blots are presented in Supplementary Fig. S9. The treatment groups were Control (untreated); IR, (75 Gy irradiation); IR + PM014 (IR + 200 mg/kg PM014); PM014 (200 mg/kg PM014 only). (**D**) The pro-apoptotic protein Noxa (green) was co-stained with pro-SP-C (red), a marker for type II AECs, using irradiated mouse lung tissue. Magnification × 400 (Scale bar, 50 μm).

To confirm the induction of apoptosis by IR, we performed an in situ terminal deoxynucleotidyl transferase dUTP nick-end labeling (TUNEL) assay using irradiated lung tissue. Radiation increased the number of apoptotic nuclei in lung tissue and this degree of apoptosis was decreased by PM104 treatment (Fig. 5A, Supplementary Fig. S5). The pro-apoptotic protein Noxa is reportedly involved in radiation-induced lung fibrosis^22^. Immunohistochemistry and western blotting showed higher expression levels of Noxa in irradiated lungs than in control lungs, and that PM014 attenuated these IR-induced increases in Noxa expression (Fig. 5B,C). Noxa levels were increased and co-localized with pro-SP-C in epithelial cells from the irradiated lung tissue, which was reduced by PM014 (Fig. 5D, Supplementary Fig. S6). These results suggest that PM014 reduces the expression of Noxa and attenuates epithelial cell death, resulting in inhibition of fibrosis.

### PM014 suppresses fibrosis in orthotopic lung tumours in combination with radiation

**Figure 6:**
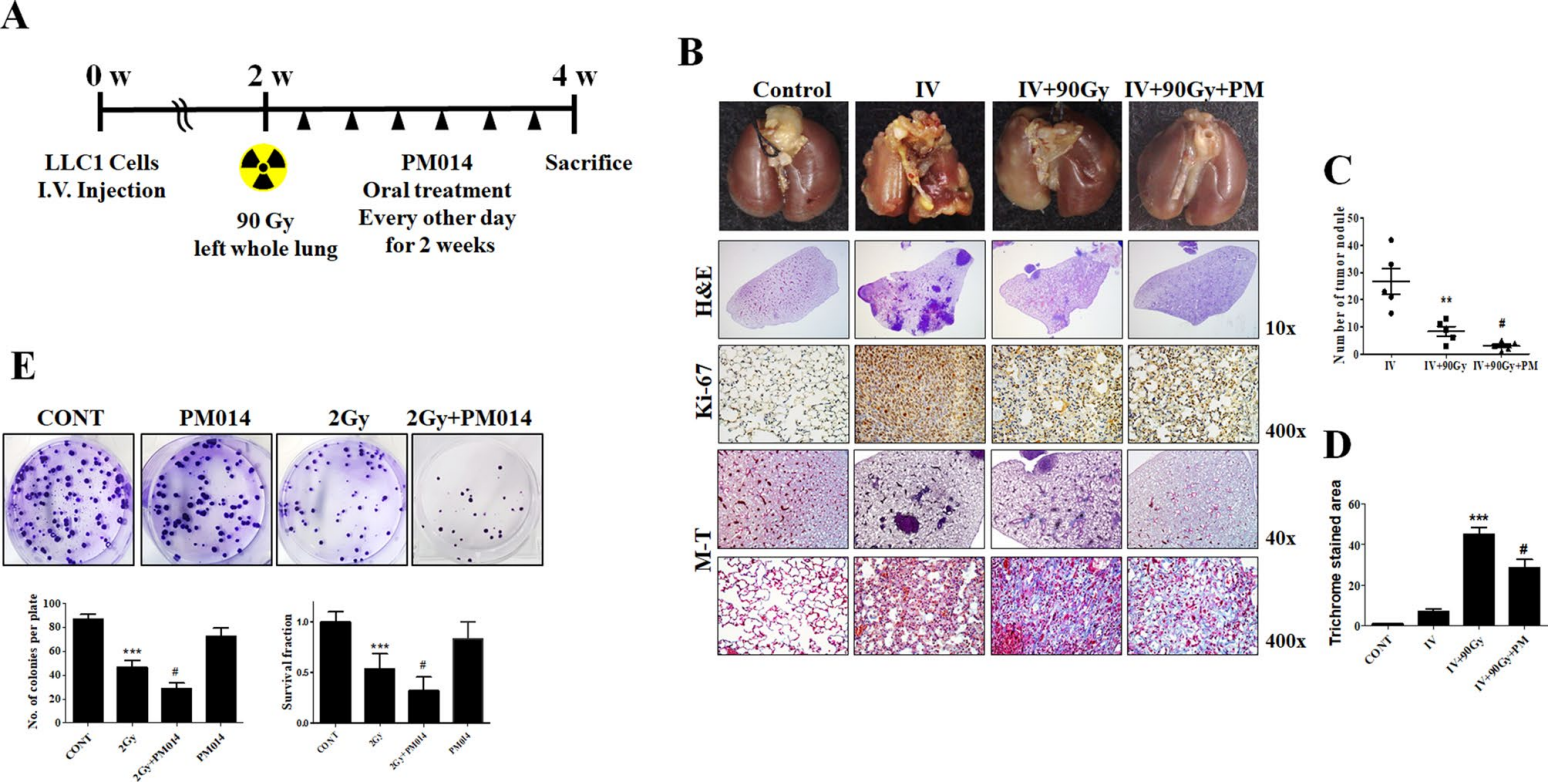
PM014 improves radiotherapy in an orthotopic lung tumour mouse model. (**A**) Experimental scheme of the orthotopic lung cancer model. (**B**–**D**) Gross (top), H&E (2nd from the top), Ki-67 (middle), and Masson’s Trichrome (M-T, 2nd from the bottom and the bottom) results using the orthotopic lung cancer mouse model. **P < 0.01 vs. IV; ^#^P < 0.05 vs. IV + 90 Gy. (**E**) Colony-forming activity was examined in H460 cancer cells. The treatment groups were Control (untreated); PM014 (10 mg/mL PM014 only); IR, (2 Gy irradiation); IR + PM014 (IR + 10 mg/mL PM014). ***P < 0.05 vs. 2 Gy; ^#^P < 0.01 vs. 2 Gy + PM014.

To elucidate the effect of using PM014 in combination with radiotherapy of lung tumours, we established an orthotopic lung tumour model after IV injection with LLC1 cells. The left whole lung of the orthotopic tumour model was irradiated with 75 Gy. However, this 75 Gy irradiation led to fibrosis after 4 weeks, and the size of the orthotopic lung tumour was so large that the mice died. Therefore, the radiation dose was increased to 90 Gy, and a 2-week model was also chosen for the study (Supplementary Fig. S7). The experimental schedule is shown in Fig. 6A. In the orthotopic lung tumour model, there were 40–50 visible tumour nodules per lung. After irradiation, the number of nodules was dramatically decreased compared to that in IV only mice. Interestingly, tumour regression was greater in mice irradiated with 90 Gy and treated with PM014 (Fig. 6B,C), with the result being almost similar to that for control mice. Fibrosis was significantly increased in irradiated normal lung lesions, which was greatly reduced in mice treated with IR and PM014 (Fig. 6B,D). A colony formation assay was performed to confirm whether PM014 synergizes with IR. PM014 caused the lung cancer cells to become more sensitive to radiation (Fig. 6E). In this study, we treated A549 lung cancer cells cultured under sphere forming conditions with PM014 (0, 5, and 10 μg/mL) to study the effects of PM014 on lung cancer stem-like cells. We found that PM014 suppressed sphere formation in this lung cancer cell line (Supplementary Fig. S8), suggesting that PM014 may have the potential to suppress cancer stem-like cells. However, the effect of PM014 on cancer stem cells only suggests the possibility and further research is needed. Taken together, these results suggest that PM014 treatment in combination with radiotherapy inhibits the fibrosis of normal tissue without interfering with tumour removal and is a promising therapeutic strategy for controlling IR-induced lung fibrosis.

## Discussion

To study the radiation-induced damage of normal tissues adjacent to tumours that underwent radiotherapy, we previously established an in vivo mouse model that mimicked clinical SBRT^23,24^. Using this model, inflammatory responses of the irradiated lung were studied at 2 weeks after irradiation with 75 Gy^8^, and fibrosis could be clearly observed at 6 weeks.

PM014 comprises seven major herbal components found in Chung-Sang-Bo-Ha-Tang, a well-known herbal mixture for treating pulmonary diseases in traditional Korean medicine^8,25^. The optimal dosing and timing of PM014 administration to treat radiation-induced lung injury have been previously assessed in our laboratory, with the optimal conditions being 200 mg/kg, 3 times a week^8^. Six weeks after irradiation, an excessive deposition of collagen was markedly attenuated in the mice treated with PM014, suggesting that PM014 has antifibrotic potential (Fig. 1f,g). These results are consistent with the reduction of collagen by PM014 in bleomycin induced pulmonary fibrosis model^26^. Immunohistochemistry to assess 8-OHdG and NOX4 levels suggested that PM014 has antioxidant properties. It is reported that expression of nox4 is increased by irradiation and blocking of nox4 is attenuated irradiation induced fibroblast differentiation^27^. Our results have shown a significant decrease in the expression of NOX4 by PM014. Thus, PM014 is an effective drug for reducing irradiated mediated fibroblast differentiation. In our previous study, we identified a new molecular mechanism underlying IR-induced fibrosis by demonstrating that Noxa-induced apoptosis plays an important role in fibrosis^22^. As shown in Fig. 5, PM014 attenuated IR-mediated apoptosis and Noxa upregulation in AECs. These results suggest that PM014 reduces IR-induced fibrosis by inhibiting Noxa expression and apoptosis in AECs. IR-induced apoptosis mainly occurs through DNA damage induction^28^. To investigate the role of PM014 in IR-induced DNA damage, we assessed γH2AX levels and performed a comet assay^29^. Following irradiation, the levels of γH2AX and the length of the comet tail were increased in irradiated cells, but were, importantly, decreased in PM014 treated cells, suggesting that radiation injury can be exacerbated by DNA damage of lung epithelial cells and that PM014 protects these cells from radiation-induced DNA damage, thereby inhibiting fibrosis. However, whether PM014 regulates DNA repair is unclear; thus, further studies are needed to address whether PM014 is related to DNA repair and fibrosis.

IR is known to activate the NF-κB pathway^30^, and *IL-6* and *Twist* are downstream molecules in the NF-κB pathway. Therefore, PM014 may play a role in suppressing the NF-κB pathway including the *IL-6* and *Twist* genes, which are some of the major regulators of IR-mediated EMT progression. Our results showed that PM014 attenuated the activation of the NF-κB promoter and the nuclear translocationt of NF-κB induced by IR (Fig. 3E–G). These results suggest that inhibition of NF-κB activation by PM014 can lead to reduced expression of downstream molecules, thereby inhibiting IR-mediated EMT progression. Our results showed that PM014 reduced the expression of TGF-β1. The transforming growth factor β1 (TGF-β1) is reported to be an important cytokine for the process of fibrosis through the interaction between growth factors and cytokines^31–36^. After irradiation, TGF-β gene expression increases, and TGF-β strongly promotes collagen synthesis and expresses collagen and fibronectin synthesis genes in fibroblasts^37–40^. Several studies have been reported to inhibit lung damage caused by radiation using a small molecule that inhibits TGF-β^41,42^. Also, TGFβ1 Stimulates Connective Tissue Growth Factor (CTGF) Expression^43^. CTGF is known as the central mediator of tissue remodeling and fibrosis and is being studied as a target that can inhibit the fibrosis process^44^. PM014 is likely to act on various targets related to fibrosis by inhibiting TGF-β.

The results of the micro-CT and flexiVent analyses in the present study correlated with the histopathological findings, suggesting a critical role for PM014 in lung fibrosis and that PM014 may be an effective therapeutic agent for inhibiting IR-induced lung fibrosis.

In the orthotopic lung cancer mouse model, most of the tumour was removed by IR compared with the large tumour nodules observed in IV only mice; however, fibrosis was observed in the irradiated area. PM014 treatment in IR-treated mice resulted in a more effective regression of the tumour and an attenuation of fibrosis. These results show that PM014 has a beneficial effect on radiation-induced fibrosis of normal tissue in an irradiated orthotopic lung cancer model. One important factor that needs to be taken into consideration in developing a drug that inhibits the fibrosis that occurs after tumour radiotherapy is whether the drug interferes with the tumour removal by irradiation, and whether it has any effect on tumour recurrence. We hypothesise that PM014 can be effectively used in radiotherapy by attenuating radiation induced fibrosis while not attenuating the efficacy of the radiation therapy.

In conclusion, our results collectively show that PM014 may be effectively and safely used as a therapeutic drug for use in combination with radiotherapy to treat lung cancer and could also act as a radioprotector for lung tissue by attenuating pneumonitis and fibrosis.

## Methods

### Micro-computed tomographic analysis

Micro-computed tomography (CT) images were acquired using a volumetric CT scanner (NFRPolaris-G90MVC: NanoFocusRay, Iksan, South Korea) at 50 kVp, 180 µA, and 150 mGy (number of views, 700; frame rate, 142 ms)^8^. Images were reconstructed (image size, 1232 × 1120 pixels; number of slices, 512) by volumetric cone-beam reconstruction (Feldkamp-Davis-Kress method) in in-line/off-line modes. Volumetric analysis was performed using the Image J software. In order to minimise inter-specimen variations in measurement, identical level settings were used for analysis of all images.

### Functional assessment of the lungs

Lung function in irradiated mice was evaluated with the flexiVent system (flexiVent; SCIREQ, Montreal, QC, Canada), which measures flow-volume relationships in the respiratory system, including forced oscillation, to discriminate between airway and lung tissue variables^17^. Evaluations were performed according to the manufacturer’s instructions. Briefly, after anesthetisation, mice were connected to a computer-controlled small-animal ventilator and quasi-sinusoidally ventilated with a tidal volume of 10 mL/kg at a frequency of 150 breaths/minute. Measurement commenced when a stable ventilation pattern without obvious spontaneous ventilator effort was observed at the ventilation pressure tracing. All perturbations were performed sequentially until three acceptable measurements (coefficient of determination > 0.95) were recorded for each subject, from which an average was calculated.

### RT-PCR and western blotting

RNA was isolated from lung tissues of mice by RNeasy Mini Kit (Qiagen, CA, USA), according to manufacturer’s instructions. Real-time reverse transcription-polymerase chain reaction (RT-PCR) was performed using Light Cycler 480 SYBR Green I master mix and Light Cycler 480 real-time PCR machine (Roche Applied Science, Indianapolis, IN, USA)^8^. Quantification was performed by comparative CT method (∆∆CT). Extracted 30 μg proteins were separated by SDS-PAGE, the membranes were probed with primary antibody (1:1000), followed by incubation with horseradish peroxidase-coupled secondary antibody^45^. Detection was performed with a chemiluminescence-based detection kit (Bio-Rad, Hercules, CA, USA).

### Preparation of PM014

PM014 comprises seven species of herbal extracts as described in previous study^8^ (Supplementary Table S2) and has been approved for the Investigational New Drug (IND) program by the Ministry of Food and Drug Safety, Republic of Korea (ID:20130030575).

### Preparation of lung tissues for histology and immunohistochemistry

Left-lung tissues of irradiated mice were fixed in 4% paraformaldehyde and then dehydrated and embedded in paraffin. For histological study, 4 μm tissue sections were stained with haematoxylin and eosin (H&E), Massons trichrome (MT) and immunohistochemical (IHC) stains^8^. For detection of TGF-β1, tissue sections were incubated with an anti-TGF-β1 primary antibody (1:100 dilution; ab64715, Abcam) at 4 ℃ overnight. Slides were then incubated with avidin^18^. Slides were then incubated with avidin–biotin peroxidase complex (ABC kit, Vector Laboratories, CA, USA) 4715, Abcam) and were developed using 3,3′-diaminobenzidine tetrachloride (DAB; Zymed Laboratories, CA, USA).

### Histology and immunohistochemistry evaluation

Slides were assessed according to a dual-rate semi-quantitative method by three independent pathologists, who were blinded to sample identities^46^. For histological evaluation, lung tissue sections were stained with H&E and MT staining and scored for the number of inflammation or fibrotic foci, respectively. For IHC evaluation, lung tissue sections were stained with TGF-β1 staining. Randomly selected fields of each slide were scored for area and intensity of positively stained (brown) cytoplasm and cell membrane. Intensity scores were assigned as follows: 0 = no appreciable staining (negative); 1 = barely detectable staining (weak); 2 = readily appreciable brown staining (moderate); and 3 = dark brown staining (strong positivity). The total score was calculated by adding the intensity scores from five independent views in each sample, resulting in a final score of 0 to 15. For statistical analysis, scores 3–15 and 0–2 were defined as indicating positive and negative expression, respectively.

### Immunocytochemistry

Cells were cultured on coverslips coated with poly-_L_-lysine, fixed with 4% paraformaldehyde in PBS and permeabilised with 0.1% Triton X-100^22^. The cells were incubated with primary antibodies at 4 °C overnight, and then stained with secondary antibodies. These cells were viewed by confocal microscopy (LSM 700, Zeiss, Jena, Germany).

### Luciferase assay

The pGL4.32[luc2P/NF-κB-RE/Hygro] plasmid containing five copies of an NF-kB response element was purchased from Promega (Promega Corp., Madison, WI, USA). Luciferase activity was measured in samples containing equivalent amounts of protein using a luminometer and luciferase assay reagents.

### DPPH radical scavenging assay

Microplate 2, 2-diphenyl-1-picrylhydrazyl (DPPH) assay was performed^47^. Briefly, in a 96 well plate, sample dilutions (standard stocks of different samples 5 mM) in triplicate received 40 μM DPPH solution, and absorbance was measured at 550 nm using a microplate reader.

### TUNEL assay

TUNEL assays were performed using the TUNEL Assay Kit (ab206386) (abcam, Cambridge, United Kingdom) according to the manufacturer’s protocol. Briefly, Briefly, terminal deoxynucleotidyl Transferase binds to exposed 3′-OH ends of DNA fragments generated in response to apoptotic signals and catalyzes the addition of biotin-labeled deoxynucleotides and biotinylated nucleotides are bound with a streptavidin–horseradish peroxidase (HRP) conjugate.

### Cell culture

The human normal lung epithelial cell line L132, the human lung carcinoma cell lines A549 and NCI-H460, the mouse lung epithelial cell line MLE-12 were cultured in RPMI (Gibco, Gaithersburg, MD, USA) supplemented with 10% fetal bovine serum (Gibco) in a 37 °C incubator with 5% CO_2_. Lewis lung carcinoma cell line LLC1 was cultured in DMEM (Gibco) supplemented with 10% fetal bovine serum in a 37 °C incubator with 5% CO_2_.

### Colony formation assay

A total of 500 H460 irradiated 2 Gy were seeded into a 60 mm dish and cultured in RPMI supplemented with 10% FBS for about 2 weeks. Then, the cells were fixed and stained with acetic acid: methanol (1:3) solution for 5 min, followed by staining with 0.5% crystal violet solution. The colonies were quantified using the NIH Image J program.

### Invasion assay and wound closure assay

Invasion assays were performed using the Chemicon Cell Invasion Assay Kit (Millipore, Billerica, MA, USA) according to the manufacturer’s protocol. Briefly, L132 cells (1 × 10^4^) were plated onto a Matrigel-coated transwell invasion chamber and incubated at 37 °C for 24 h. Invading cells were then fixed with methanol and stained with haematoxylin. On average, five random fields were counted using a light microscope. For Wound closure assay, when L132 cells were confluent, a wound was created along the middle of the culture plate using a tip. Cells were then irradiated (10 Gy) with or without PM014 (10 μg/mL) for 48 h and cells images obtained using inverted microscopy.

### Comet assay

Alkaline comet assays were performed using CometAssay kit (4250-050-K, Trevigen) protocol following the manufacturer’s instructions. Cells were irradiated (10 Gy) with or without PM014 (10 μg/mL). After electrophoresis, the cells were stained with diluted SYBR Green and all images were captured by fluorescence microscopy. DNA damage was quantified for at least 50 randomly selected cells by measuring tail length using the NIH Image J program.

### Orthotopic mice model

Mouse lung carcinoma LLC1 cells (1 × 10^6^) in 200 µL of physiological saline were injected into the tail vein of 7-week-old male C57BL/6 mice. Two weeks later, a single dose of 90 Gy was delivered to the left whole lung using an image-guided small-animal irradiator. The mice were then randomly divided into three groups: (1) LLC1 group—i.v. injection only group; (2) LLC1 + IR group—mice were exposed to a single dose of 90 Gy delivered to the left whole lung 2 weeks after i.v. injection; (3) LLC1 + IR + PM014 group—the mice were orally administered PM014 (200 mg/kg) for 2 weeks on every other day after irradiation. On week 4, the mice were sacrificed by CO_2_ asphyxiation, and lung tissues were collected for analysis.

### Spheres culture

A549 human lung cancer cells were resuspended in RPMI containing 20 ng/mL of epithelial growth factor (EGF), basin fibroblast growth factor (bFGF), and B27 serum free supplement as sphere forming condition.

### Statistical analysis

Statistical analysis was performed using Prism 5 software (Graph Pad Software Inc., San Diego, CA, USA). Data are presented as mean ± SE. Comparison of variables between the control and radiation-treatment groups was performed by one-way analysis of variance, followed by the Newman–Keuls multiple comparison test. p < 0.05 was considered statistically significant. In each figure, *P < 0.05, **P < 0.01 or ***P < 0.001 vs. control; ^#^P < 0.05, ^##^P < 0.01 or ^###^P < 0.001 vs. IR.

### Animal experiments

All the animal experiments were approved by the Institutional Animal Care and Use Committee of Yonsei University College of Medicine (IACUC approval number: 2019–0,147) and were performed in strict accordance with the Guide for the Care and Use of Laboratory Animals. Male C57BL/6 mice (6 weeks of age) were purchased from Charles River Korea (Orient Bio, Seongnam, South Korea). Mouse irradiation was performed using an image-guided small-animal irradiator (X-RAD 320; Precision, North Branford, CT, USA) equipped with a collimator system as described in our previous study^8^. During irradiation, mice were anesthetized with an intraperitoneally administered mixture of 30 mg/kg Zoletil (tiletamine and zolazepam) and 10 mg/kg Rompum (xylazine). The mice were randomly divided into six groups (n = 5 per group) as follows: (1) control group; (2) irradiation (IR)—mice were exposed to a single dose of 75 Gy delivered to the left lung; (3) IR + PM014—200 mg/kg of PM014 were administered orally on alternate days for 6 weeks after irradiation; (4) IR + amifostine (AMI)—100 mg/kg of amifostine were intraperitoneally administered on every other day for 6 weeks after irradiation; (5) PM014—PM014 was administered orally without irradiation. For administration, the PM014 extract was dissolved in PBS. Amifostine was prepared 10 times DMSO stock and diluted with saline for intraperitoneal injection. On week 6, the mice were sacrificed and lung tissues were prepared for analysis.

## Supplementary information

Supplementary Figures

Supplementary Tables
