# Supplementary material for: PM014 attenuates radiation-induced pulmonary fibrosis via regulating NF-kB and TGF-b1/NOX4 pathways
Source: Sci Rep. 2020 Sep 30;10:16112. doi: 10.1038/s41598-020-72629-9 (PMC7527517; doi:10.1038/s41598-020-72629-9)

**PM014 attenuates radiation-induced pulmonary fibrosis via regulating NF-kB and TGF-b1/NOX4 pathways**

**Sung-Hyo Park^a^**^†^**, Jee-Youn Kim^a^**^†^**, Jin-Mo Kim^a^, Byeong Rok Yoo^a^, Song Yee Han^a^, Yoo Jin Jung^a^, Hyunsu Bae^b*^, Jaeho Cho^a^**

^a^Department of Radiation Oncology, Yonsei University College of Medicine, Seoul, Republic of Korea; ^b^Department of Science in Korean Medicine, Graduate School, Kyung Hee University, Seoul, 02447, Republic of Korea.

**Supplementary Information**

**Supplementary Figure S1.**

**
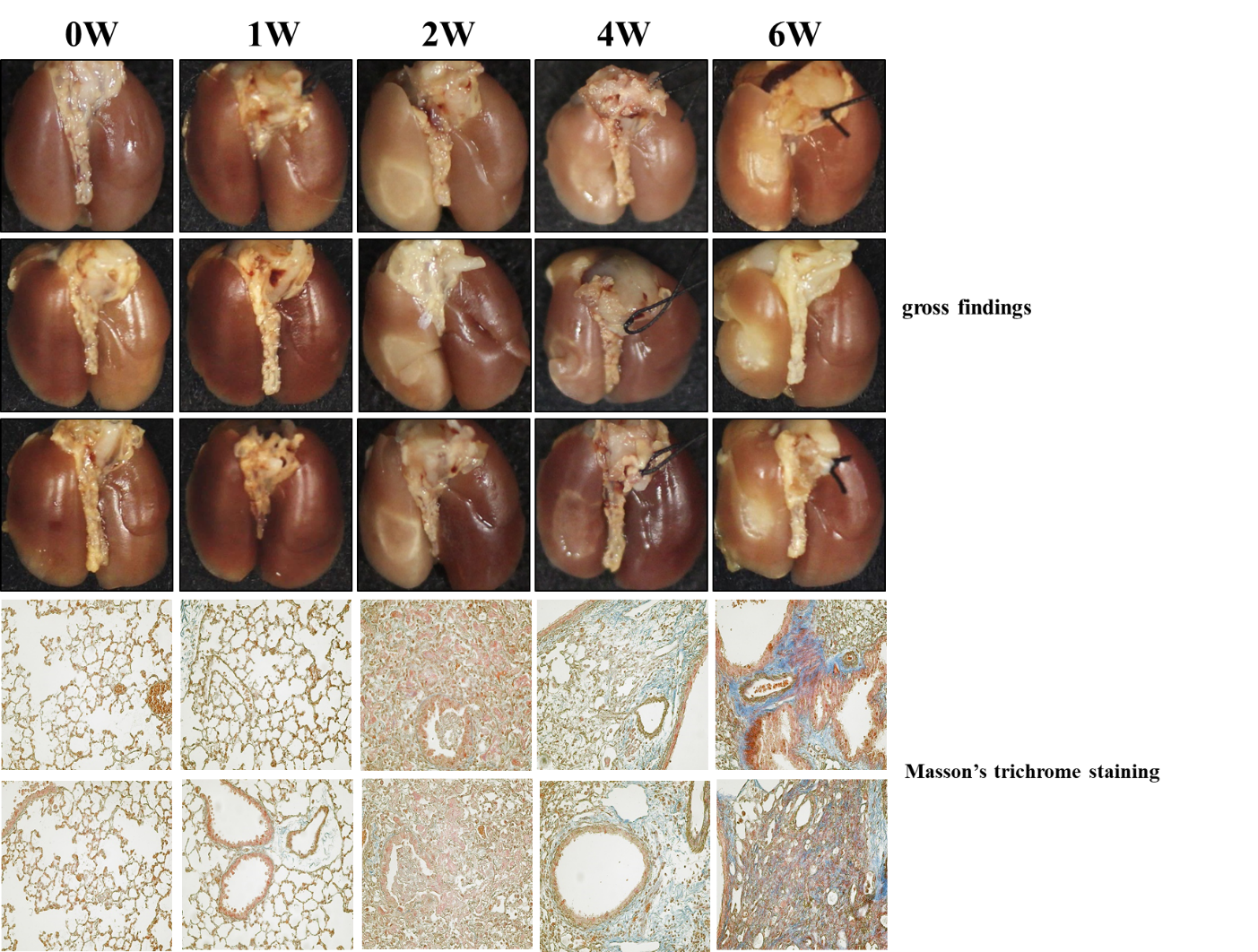
**

**Supplementary Figure S1**. Morphological change and collagen content of lungs over time by 75 Gy

Mice were sacrificed at indicated time point after 75 Gy irradiation. Lungs were immersed in fixation solution and photographed after complete fixation. To confirm fibrosis, lung tissue sections were stained with MT staining

**Supplementary Figure S2.**


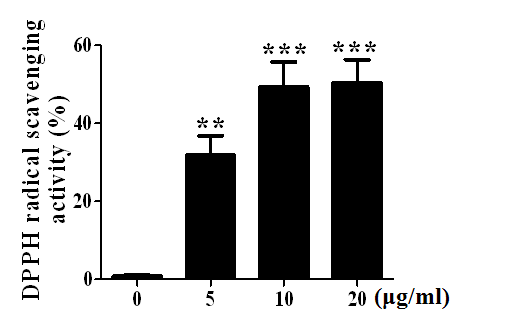


**Supplementary Figure S2**. Effect of PM014 on DPPH radical scavenging activity. PM014 exhibited radical scavenging activity in relatively dose-dependent manner. **P < 0.01 and ***P < 0.001 versus 0.

**Supplementary Figure S3.**

**Supplementary Figure S3.** Effect of PM014 on radiation-induced DNA damage. Quantification of gamma-H2AX foci numbers per cell is performed manually using the Image J program. L-132 cells were treated with 10 Gy. The treatment groups were Control (untreated); IR, (10 Gy irradiation); IR + PM014 (IR + PM014); PM014 (PM014 only).

**Supplementary Figure S4.**

**Supplementary Figure S4.**

Quantification of western blot was performed through the b-actin normalization method. L-132 cells were treated with 10 Gy. The treatment groups were Control (untreated); IR, (10 Gy irradiation); IR + PM014 (IR + PM014); PM014 (PM014 only).

**Supplementary Figure S5.**

**Supplementary Figure S5.** Quantification of TUNEL staining of mouse lung tissues using the Image J program. The treatment groups were Control (untreated); IR, (75 Gy irradiation); IR + PM014 (IR + 200 mg/kg PM014); PM014 (200 mg/kg PM014 only).

**Supplementary Figure S6.**

**Supplementary Figure S6.** Quantification of Noxa and pro-SP-C staining using the Image J program. The treatment groups were Control (untreated); IR, (75 Gy irradiation); IR + PM014 (IR + 200 mg/kg PM014); PM014 (200 mg/kg PM014 only).

**Supplementary Figure S7.**


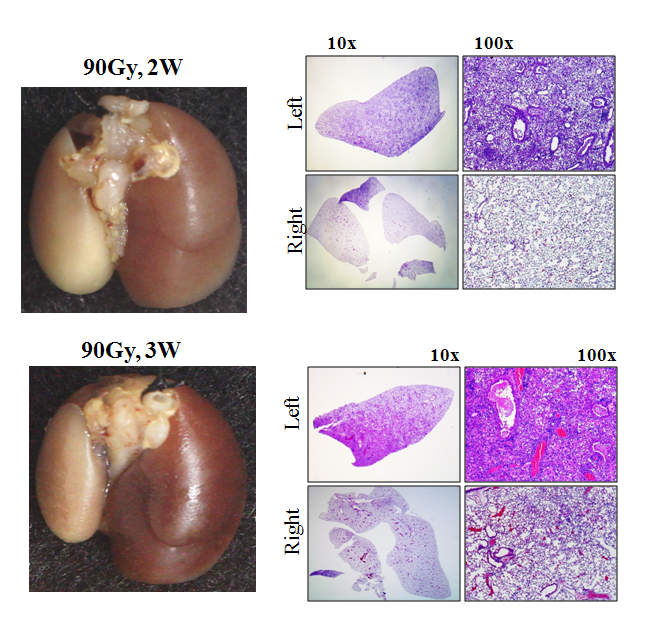


**Supplementary Figure S6**. Morphological change of lungs over time by 90 Gy

A single dose of 90Gy was delivered to the left whole lung using an image-guided small-animal irradiator. Mice were sacrificed at 2 or 3 weeks after irradiation. Lungs were photographed after complete fixation (left) and lung sections were stained with haematoxylin and eosin (right). Magnification, 10x, 400x

**Supplementary Figure S8.**


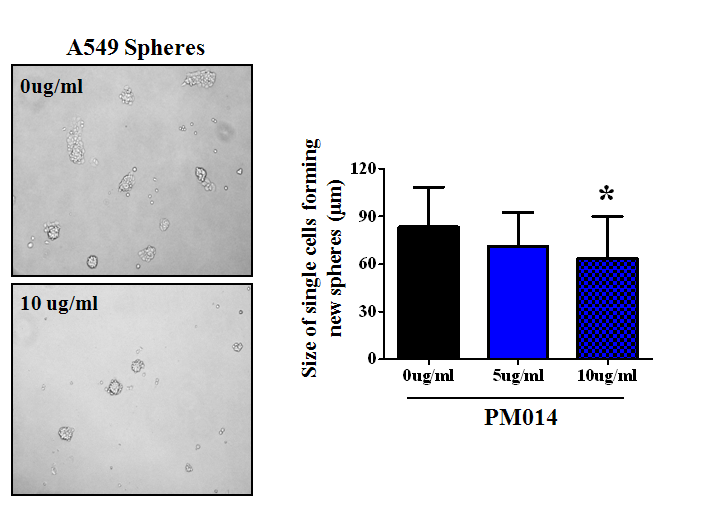


**Supplementary Figure S8**. Effect of PM014 on spheres formation in lung cancer stem-like cells. Decrease of spheres size at 7 d after PM014 treatment of sphere-cultured A549 lung cancer cells. Representative images (left) and the number of spheres (right). *P < 0.05 vs. 0

**Supplementary Figure S9.**

Uncropped western blot images


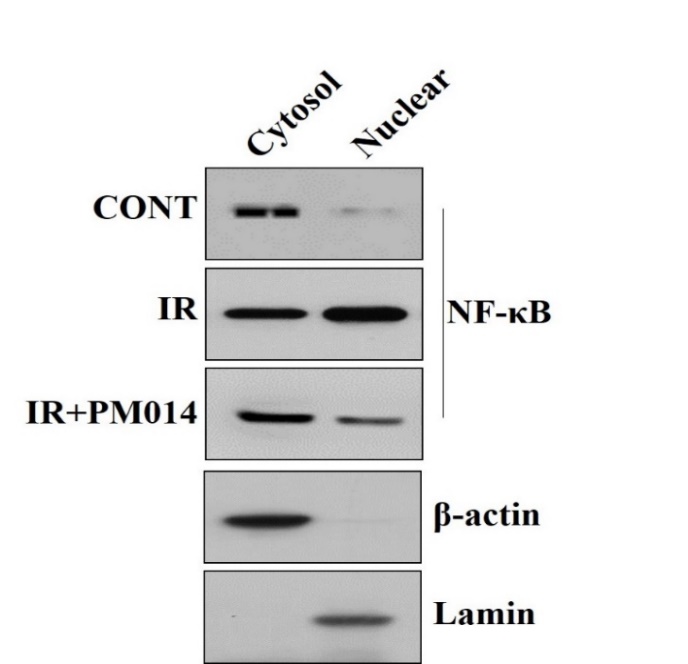
Figure 2G.


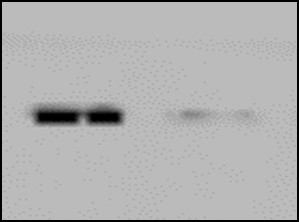


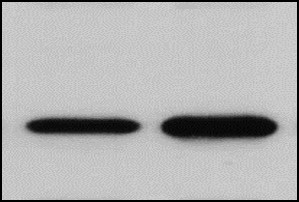


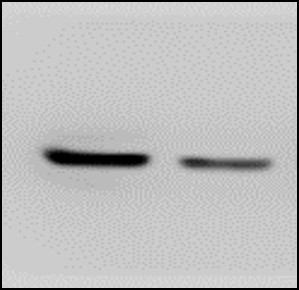


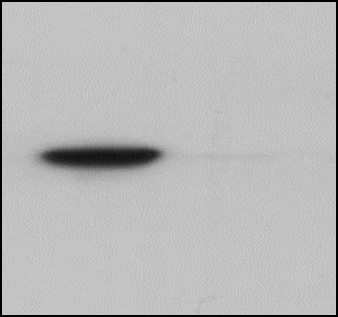


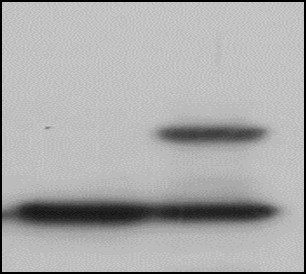


Uncropped western blot images

Figure 3C


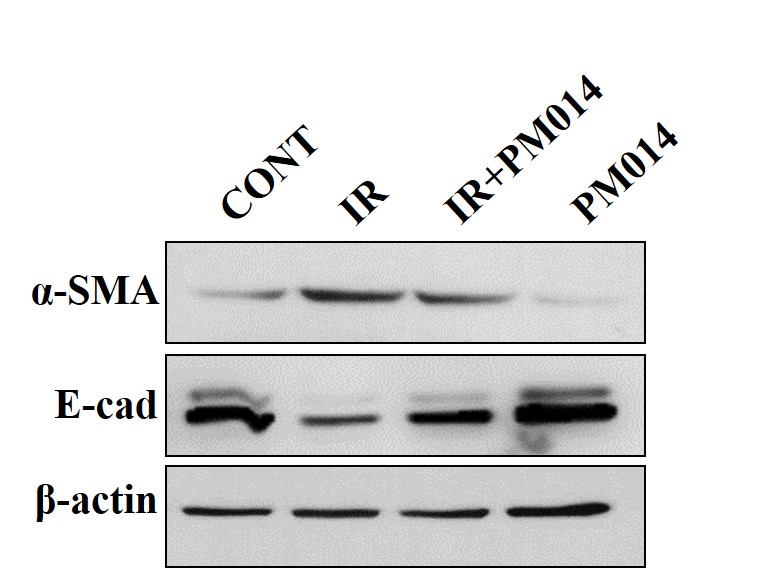

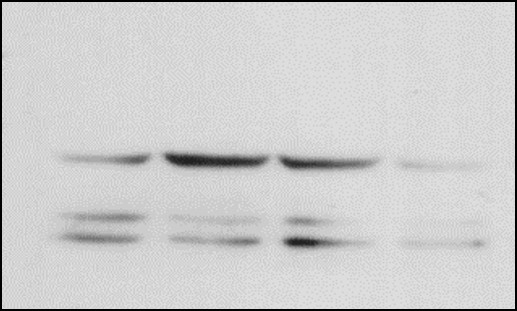


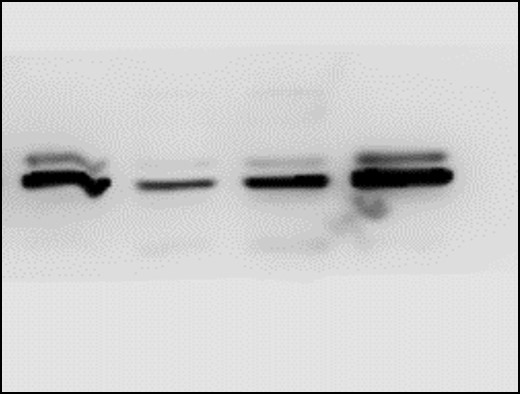


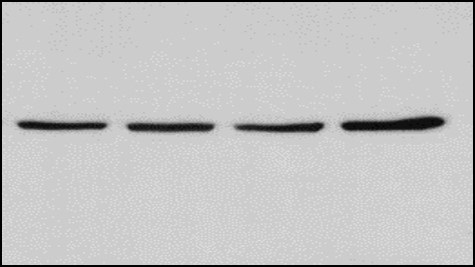


Uncropped western blot images

Figure 4D


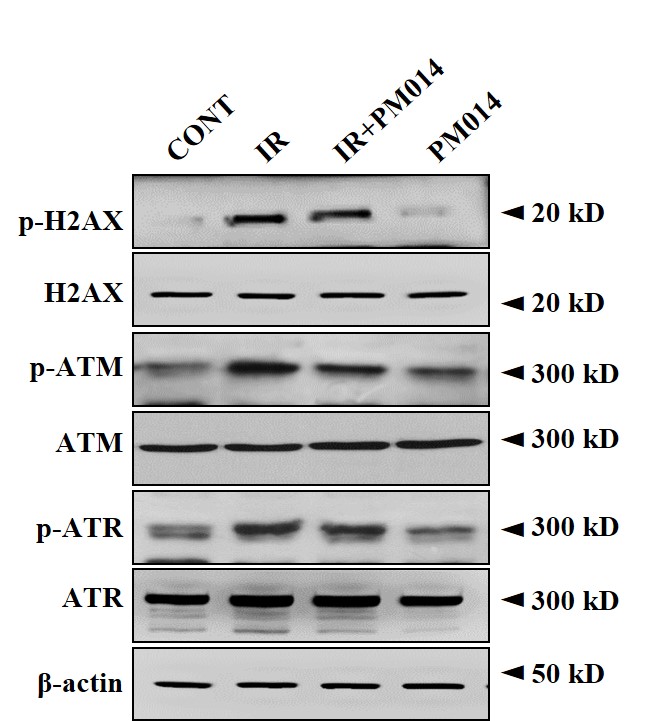


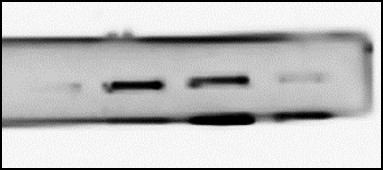


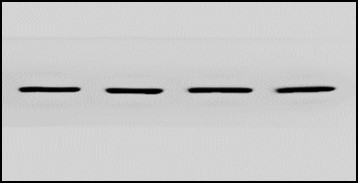


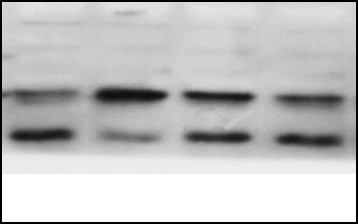


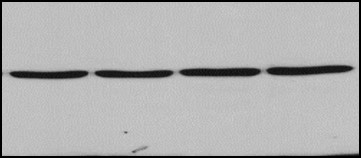


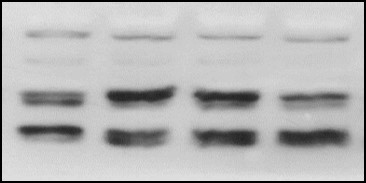


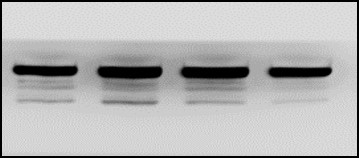


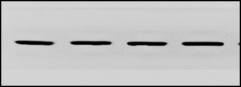


Uncropped western blot images

Figure 5C


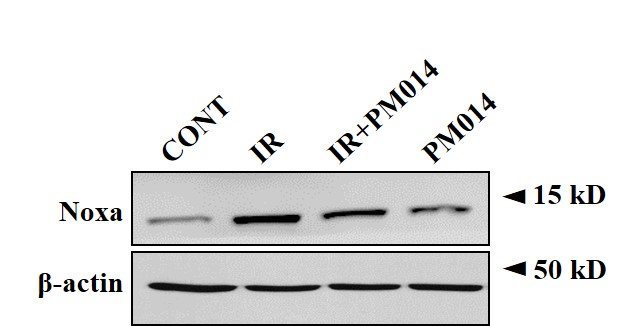

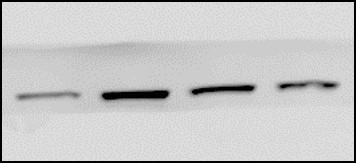

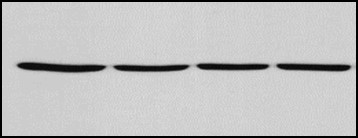

Supplement: Supplementary file 1 — Supplementary Figures [file 41598_2020_72629_MOESM1_ESM.docx]
