# Supplementary material for: PM014 attenuates radiation-induced pulmonary fibrosis via regulating NF-kB and TGF-b1/NOX4 pathways
Source: Sci Rep. 2020 Sep 30;10:16112. doi: 10.1038/s41598-020-72629-9 (PMC7527517; doi:10.1038/s41598-020-72629-9)
Supplement: Supplementary file 2 — Supplementary Tables [file 41598_2020_72629_MOESM2_ESM.docx]

**PM014 attenuates radiation-induced pulmonary fibrosis via regulating NF-kB and TGF-b1/NOX4 pathways**

**Sung-Hyo Park^a^**^†^**, Jee-Youn Kim^a^**^†^**, Jin-Mo Kim^a^, Byeong Rok Yoo^a^, Song Yee Han^a^, Yoo Jin Jung^a^, Hyunsu Bae^b*^, Jaeho Cho^a^**

^a^Department of Radiation Oncology, Yonsei University College of Medicine, Seoul, Republic of Korea; ^b^Department of Science in Korean Medicine, Graduate School, Kyung Hee University, Seoul, 02447, Republic of Korea.

**Supplementary Table**

**Supplementary Table S1. Microarray results of *TGF-β1, IL-6, Twist***

| **Antibody**  **name** | **Gene**  **symbol** | **Gene name** | **Log Fold**  **Change** | **Gene bank Assession** |
| --- | --- | --- | --- | --- |
| TGF-β1 | Tgfb1 | Transforming growth factor, beta 1 | 1.821 | NM_011577 |
| IL6 | Il6 | Interleukin 6 | 3.342 | NM_031168 |
| Twist | Twist1 | Twist basic helix-loop-helix transcription factor 1 | 6.237 | NM_011658 |

The mice were irradiated with 75 Gy, and lungs were subjected to microarray analysis after 6 weeks.

**Supplementary Table S2. Composition and amount of PM014**

| **Formula of PM014** | **Amount (g)** | **Chemical marker** |
| --- | --- | --- |
| Root of *Rehmannia glutinosa* (RG) | 600 | 5-HMF |
| Cortex of *Paeonia suffruticosa* (PS) | 300 | Paeoniflorin |
| Fruit of *Schizandra chinensis* (SC) | 300 | Schizandrin |
| Root of *Asparagus cochinchinensis* (AC) | 300 | Asparagine |
| Seed of *Prunus armeniaca* (PA) | 225 | Amygdalin |
| Root of *Scutellaria baicalensis* (SB) | 225 | Baicalin |
| Root of *Stemona sessilifolia* (SS) | 150 | Stemonine |
| Total | 2,100 g |  |

Medicinal plants of the seven species that constitute PM014 were purchased from Kyung Hee Herb Pharm (Seoul, South Korea) and processed at Hanlim Pharm Co. LTD (Seoul, South Korea). The herbs were cut and mixed to a total weight of 2100 g. The mixture was extracted with purified water (2100 mL) using a reflux condenser for 3 h at 90–100°C and then filtered using a 25-μm sieve. The supernatant was concentrated at 60°C under vacuum, using an evaporator. The extracts were mixed with 260 g dry cornstarch and vacuum dried at 60 °C. For administration, the PM014 extract was dissolved in PBS. The quantities of standard materials in 1 g of the final PM014 extract were: Paeoniflorin > 0.43 mg, Schizandrin > 0.12 mg, Baicalin > 7.26 mg, and Amygdalin > 2.48 mg. Quantification of standard materials in PM014 was performed by high-performance liquid chromatography analysis. Three independent batches of each compound were analysed for obtaining triplicate data.

**Supplementary Table S3.**

**The parameter description using the flexiVent system measurements**

| **Abbreviations** | **Parameter** | **Descritption** |
| --- | --- | --- |
| IC | Inspiratory Capacity | IC is the volume difference between functional residual capacity (FRC) and total lung capacity (TLC), Also equaling tidal volume plus the inspiratory reserve volume. |
| Cst | Quasi-static Compliance | The parameter K of the Salazar-Knowles equation reflects the curvature of the upperportion |
| G | Tissue Damping | Tissue damping is closely related to tissue resistance and reflects the energy dissipationin the lung tissues. |
| H | Tissue Elastance | Theparameter H is closely related to tissue elastance and reflects the energy conservation in the lung tissues. |
| Rn | Newtonian Resistance | The Newtonian Resistance parameter of the Constant Phase Model represents the resistance of the central airway |
| Rrs | Airway constriction | Resistance of the respiratory system |

**Supplementary Table S4. Primer sequences**

|  | **Forward sequences** | **Reverse sequences** |
| --- | --- | --- |
| **GAPDH** | 5’-CAGATCCACGACGACGGACACAT-3’ | 5’-CATCACTGCCACCCAGAAGA -3’ |
| **IL-6** | 5’-TCCACGATTTCCCAGAGAAC-3’ | 5’-CCGGAGAGGAGACTTCACAG-3 |
| **TGFb-1** | 5’-GCAACATGTGGAACTCTACCAGAA-3’ | 5’-GACGTCAAAAGACAGCCACTC-3’ |
| **twist** | 5'-TTCAGACCCTCAAACTGGCG-3’ | 5’-CTGGGAATCTCTGTCCACGG-3’ |
| **a-SMA** | 5'-TCCTGACGCTGAAGTATCCG-3' | 5'-AAGGTCTCAAACATAATCTG-3' |
| **E-CAD** | 5'-AGCTTTTCCGCGCTCCTGCT-3' | 5'-GACATGGCCTCTCTCCAGGT-3 |
